# Mechanically-induced fiducial marker system for event-based synchronization of independent recording instruments

**DOI:** 10.1088/1361-6501/ae92a4

**Published:** 2026-08-12

**Authors:** Kourosh Kalayeh, John O L DeLancey, James A Ashton-Miller, J Brian Fowlkes

**Affiliations:** 1 Department of Radiology, University of Michigan, Ann Arbor, MI, United States of America; 2 Department of Urology, University of Michigan, Ann Arbor, MI, United States of America; 3 Department of Biomedical Engineering, University of Michigan, Ann Arbor, MI, United States of America; 4 Department of Obstetrics and Gynecology, University of Michigan, Ann Arbor, MI, United States of America; 5 Department of Mechanical Engineering, University of Michigan, Ann Arbor, MI, United States of America

**Keywords:** event-based synchronization, fiducial marker, high-resolution manometry (HRM), stress urinary incontinence (SUI), transperineal ultrasound (TPUS), pelvic floor disorders

## Abstract

Many experimental and clinical measurements require simultaneous acquisition from independent instruments that do not share a clock. We describe a device that enables such pairings via event-based synchronization: an electrical trigger from one instrument drives an Arduino-controlled stepper actuator that produces a brief mechanical event at a location sensed by the other instrument, leaving a fiducial marker for post-hoc temporal alignment. The motivating application is synchronization of high-resolution manometry (HRM) with transperineal ultrasound for phenotyping stress urinary incontinence. Bench characterization yielded a device latency of ${802.5}\,\unicode{x03BC}\textrm{m} \pm {46.2}\,\unicode{x03BC}\textrm{m}$ (mean $\pm$ SD, $n = 30$), of which the electromechanical response of the actuator accounts for $\approx\!{84}\%$ of the mean latency and the dominant share of the trial-to-trial variation (i.e. the SD of ${46.2}\unicode{x03BC}\textrm{m}$). For the ultrasound–HRM application, the synchronization uncertainty is $\approx\!{6}\,\textrm{ms}$ ($n = 30$ markers), set by the HRM rather than the device, whose intrinsic jitter contributes negligibly. The approach generalizes to instrument pairings in which one instrument provides a stable electrical trigger with a known relation to its recording timeline and the other is a mechanically-sensitive receiver of adequate bandwidth, provided the marker does not contaminate the signal of interest.

## 1. Introduction

Many experimental and clinical measurements require simultaneous acquisition from independent recording instruments that run on separate clocks. When both instruments expose the necessary interfaces, the cleanest solution is to link them directly — for example, via a trigger in/out port, a trigger-distribution board, data-acquisition logging of transistor–transistor-logic (TTL) triggers, an open-hardware synchronization board [1], or a software-timestamping framework such as Lab Streaming Layer [2] — so that they share a common trigger or clock and no post-hoc alignment is required. When such a link is unavailable — a common situation when the instruments are from different manufacturers, of different vintages, or run closed acquisition software that exposes no trigger port, TTL line, or programmatic access — temporal alignment must instead be recovered from features present in the recordings themselves.

Event-based synchronization addresses this by injecting a fiducial event into all recording streams that can be recovered post hoc [3]; the film clapperboard is a familiar example. Other examples include subject-performed actions such as claps, jumps, finger taps, and fist-on-table strikes [3–5], gait events such as heel-strike recovered from the recordings themselves [6], foot stomps [7], and automatically-detected shared physical events in heterogeneous sensor streams [8].

Here, the contribution is not a new synchronization principle but a custom electromechanical implementation for a pair of instruments — one providing an electrical trigger output, the other mechanically sensitive — that share no clock, trigger, or signal domain. Specifically, the shared event is a mechanical perturbation generated by an electrically-triggered actuator under the control of one of the recording instruments. Because the marker is generated by solid-state electronics and a stepper actuator, its onset jitter is set by electromechanical response. The device-side contribution to synchronization uncertainty is therefore at the microsecond scale. Because the marker is mechanical, the receiving instrument need only be mechanically sensitive — pressure transducer, strain gauge, piezoelectric sensor, accelerometer, etc — making the approach applicable to instrument pairings whose native signal domains differ. This design pattern is useful whenever four conditions are met: (i) the source instrument provides a stable electrical trigger; (ii) that trigger has a known relationship to the source instrument’s own recording timeline, so the marker can be located within the source’s data; (iii) the receiving instrument is mechanically sensitive, with bandwidth sufficient to register the marker; and (iv) the marker can be introduced without contaminating the signal of interest.

The motivation for this work is to enable cross-domain synchronization between high-resolution manometry (HRM) and transperineal ultrasound (TPUS) imaging in the context of studying pelvic-floor kinematics and urodynamic pressures as they pertain to phenotyping stress urinary incontinence (SUI) — involuntary leakage of urine during stress events (such as coughing) that increase intra-abdominal pressure. In continent subjects, the levator ani contracts in advance of the rise in intravesical pressure [9–11]. Disruption of this anticipatory activation is hypothesized as one of the potential failure mechanisms in SUI [12–14]. Quantifying anticipatory activation requires synchronized recordings of levator ani motion — visible in TPUS imaging and quantified using our prior method [15, 16] — and intravesical pressure (measured by HRM [17]). HRM samples at ${100}\,\textrm{Hz}$ and the TPUS scanner captures cine loops at 30–60 Hz on independent clocks with no shared trigger input; to our knowledge, no prior method has reported simultaneous HRM and TPUS recording with sub-frame temporal alignment.

In this study, we describe the device and its signal-conditioning chain and characterize its latency and trial-to-trial jitter on the bench. Using the ultrasound–HRM pairing as an example application, we then close the timing chain from the ultrasound trigger to the HRM marker, assemble the synchronization-uncertainty budget, and measure the ultrasound–HRM clock drift.

## 2. Materials and methods

**Figure 1.**
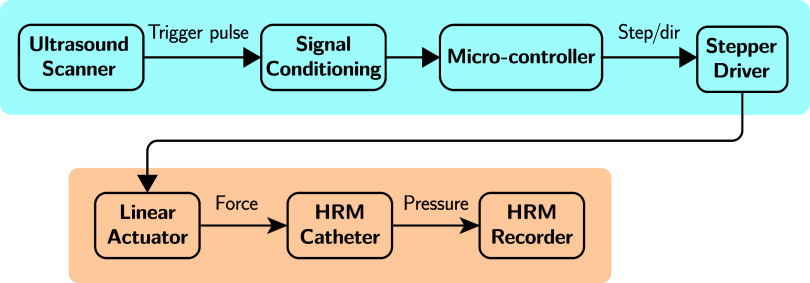
Block diagram of the full signal chain from the ultrasound scanner’s trigger output to the fiducial marker in the high-resolution manometry (HRM) recording. The device converts the electrical trigger pulse (blue region, ) into a mechanical perturbation on the HRM catheter (orange region), which is received by the HRM recorder’s native pressure sensing and appears as a transient fiducial marker in the HRM pressure trace used for post-hoc alignment. Straight arrows denote electrical signals and tapered arrows denote mechanical coupling.

**Figure 2.**
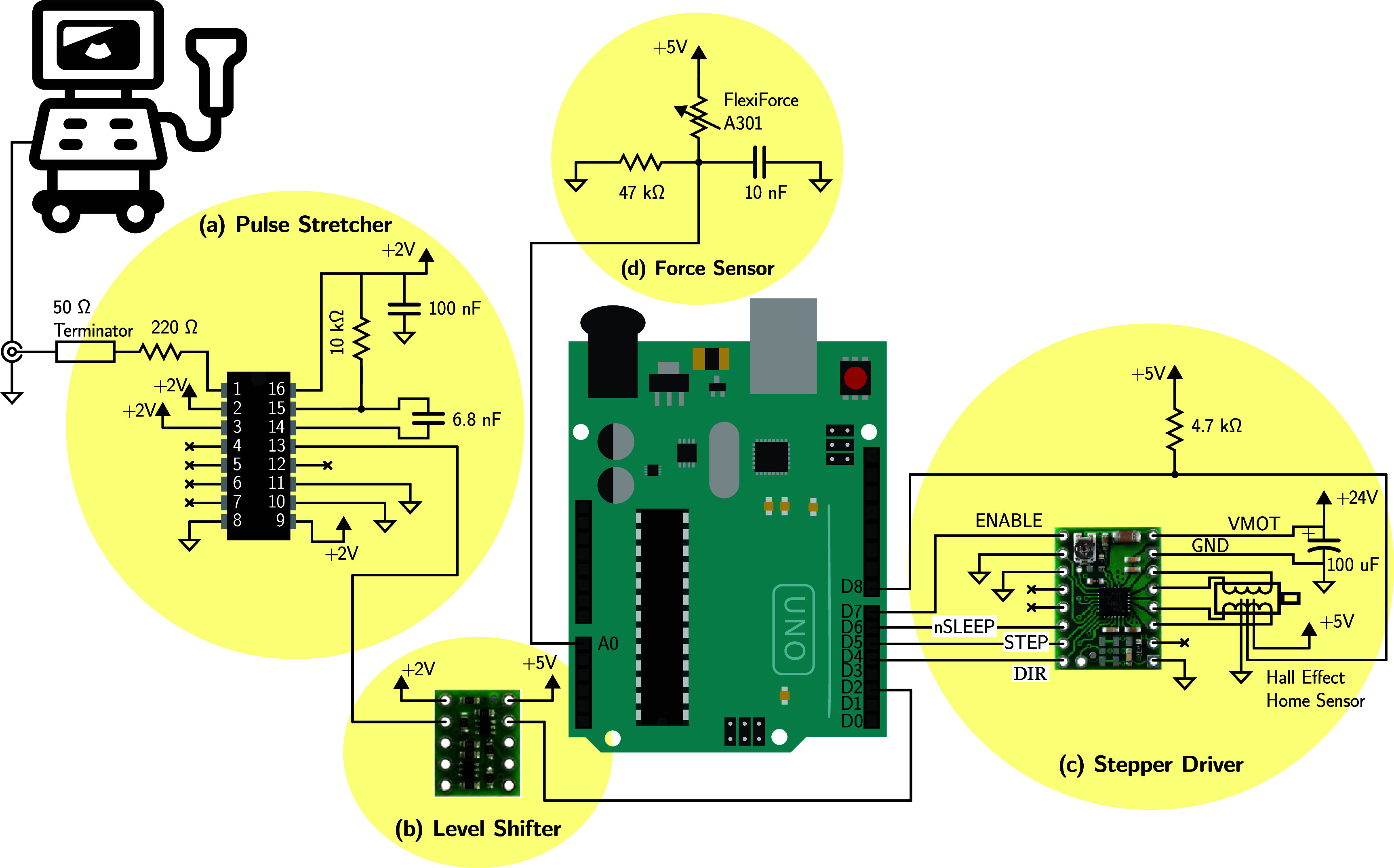
Circuit schematic of the synchronization device: (a) a pulse stretcher and (b) a level shifter that condition the brief, low-level ultrasound trigger into a clean logic edge for the Arduino, (c) a stepper driver for the MPPV-2 actuator, and (d) a FlexiForce force sensor used only for latency characterization. Component values are shown in the schematic, and the conditioning and drive stages are described in the Materials and Methods (sections 2.1 and 2.2).

The block diagram of the device’s full signal chain and the corresponding circuit schematic are shown in figures 1 and 2, respectively. As shown, the device has two functional subsystems: a stepper linear actuator that presses a piston against a location sensed by the receiver to produce the mechanical event (the fiducial marker), and a signal-conditioning chain that converts the trigger pulse from the source instrument into a clean digital edge. An Arduino Uno R3 microcontroller (Arduino, Monza, Italy), built around an ATmega328P [18], reads the conditioned trigger and drives the actuator.

### 2.1. Stepper actuator

**Figure 3.**
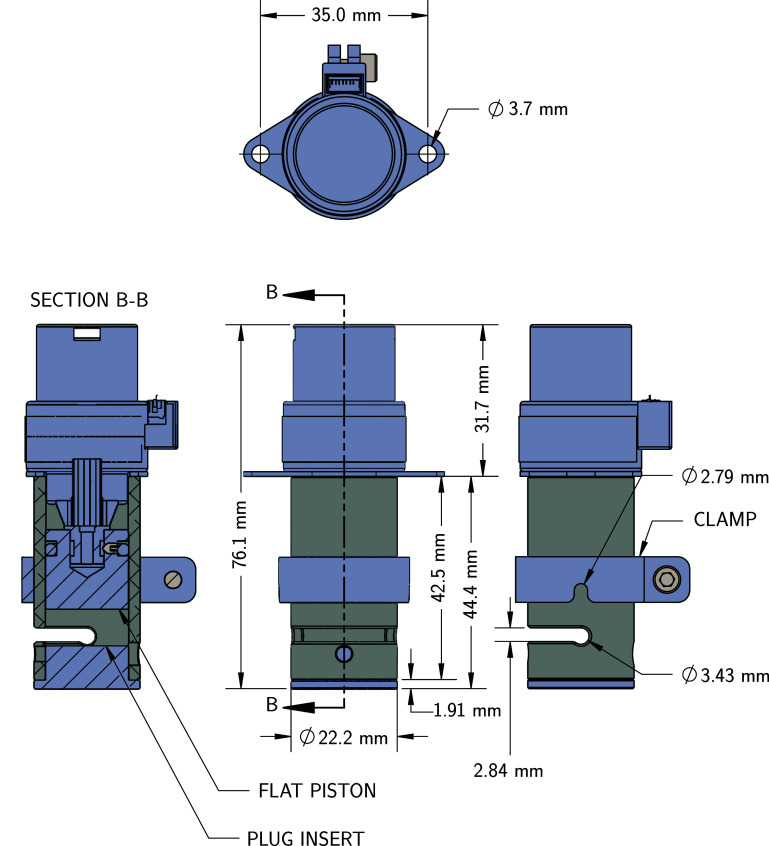
Mechanical drawing of the modified MPPV-2 stepper linear actuator (Resolution Air, Ltd), based on the manufacturer’s drawing [19]. The figure shows two modifications that Resolution Air designed and fabricated for the authors’ custom unit: a flat piston replacing the standard ridged piston, and a plug insert replacing the V-groove pinch pin, together providing uniform contact with the HRM catheter.

A proportional pinch valve with a stepper linear actuator (MPPV-2, Resolution Air, Cincinnati, OH, USA) produces the fiducial pressure marker on the HRM catheter. The MPPV-2 uses a captive leadscrew driven by a bipolar stepper motor to translate a piston with ${12.7}\,{\unicode{x03BC}\textrm{m}}$/step resolution [19]. The catheter passes through a front-loading slot; the piston presses it against a plug on the opposite side (figure 3). The standard ridged piston and V-groove pinch pin are replaced with custom flat surfaces to provide uniform contact with the catheter.

The motor is driven by a chopper-type stepper driver (DRV8434A carrier board, Pololu #3764, Pololu Corporation, Las Vegas, NV, USA) that accepts step/direction logic-level inputs from the Arduino (figure 2). The driver regulates the per-phase current to ${0.385}\,\textrm{A}$ (the motor’s rated current [19]), set by adjusting the on-board potentiometer to $V_{\mathrm{REF}} = {0.51}\,\textrm{V}$ ($I_{\mathrm{limit}} = V_{\mathrm{REF}} / 1.32$) [20]. Motor power is supplied by a separate ${24}\,\textrm{V}$ DC adapter connected to the driver’s VMOT input; a ${100}\,{\unicode{x03BC}\textrm{F}}$ electrolytic capacitor across VMOT and ground suppresses voltage transients during stepping.

The MPPV-2 includes a built-in Hall effect home sensor that provides an absolute position reference. The sensor is open-collector and requires an external ${4.7}\,\,\textrm{k}\Omega$ pull-up resistor to ${5}\,\textrm{V}$; the output reads LOW when the piston is at the home position and HIGH otherwise. On power-up, the firmware executes a homing sequence (retracting at reduced speed until the sensor triggers) to establish a known position from which subsequent piston motion is referenced.

The MPPV-2 leadscrew is non-backdrivable: the piston holds its position without motor current. After each advance–retract cycle, the firmware drives the ENABLE pin LOW to fully de-energize the motor, eliminating idle heat dissipation.

### 2.2. Signal conditioning

**Figure 4.**
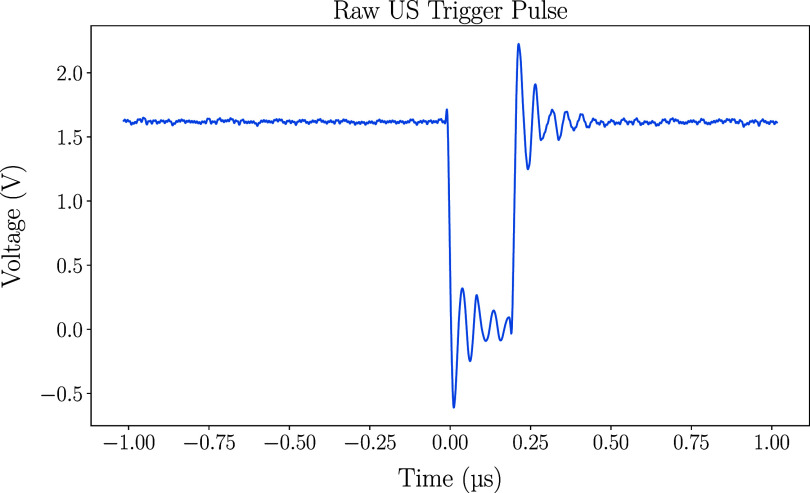
Raw ultrasound trigger pulse captured at the BNC input with the PicoScope. The ultrasound scanner (LOGIQ^®^ E10, GE HealthCare) emits a negative-logic pulse with an idle level of $\approx\!{1.6}{\,\textrm{V}}$, an active-LOW excursion to $\approx\!{0}{\,\textrm{V}}$, and a pulse width of $\approx\!{200}\,\textrm{ns}$.

Details of the signal-conditioning chain are shown in figure 2. The ultrasound scanner (LOGIQ^®^ E10, GE HealthCare, Wauwatosa, WI, USA) provides a trigger pulse output (enabled by the GE HealthCare engineering team for this work) that emits a negative-logic pulse (idle at $\approx\!{1.6}{\,\textrm{V}}$, active LOW to $\approx\!{0}{\,\textrm{V}}$) with a pulse width of $\approx\!{200}\,\textrm{ns}$ at each beam firing. A representative pulse, captured at the BNC input with a PicoScope 5244D oscilloscope (Pico Technology, St Neots, Cambridgeshire, UK), is shown in figure 4. Two problems prevent the Arduino microcontroller from directly detecting this pulse: (i) the ${1.6}{\,\textrm{V}}$ logic level lies below the ATmega328P’s $V_{\mathrm{IH}}$ threshold of ${3.0}{\,\textrm{V}}$ at ${5}{\,\textrm{V}}$ logic, and (ii) ${200}\,\textrm{ns}$ pulse width is shorter than the ATmega328P’s interrupt service routine entry latency (250–310 ns at ${16}\,\textrm{MHz}$) [18]. Therefore, the pulse needs to be stretched (figure 2(a)) and level-shifted (figure 2(b)) before reaching the Arduino input pin.

#### 2.2.1. Monostable pulse stretcher

**Figure 5.**
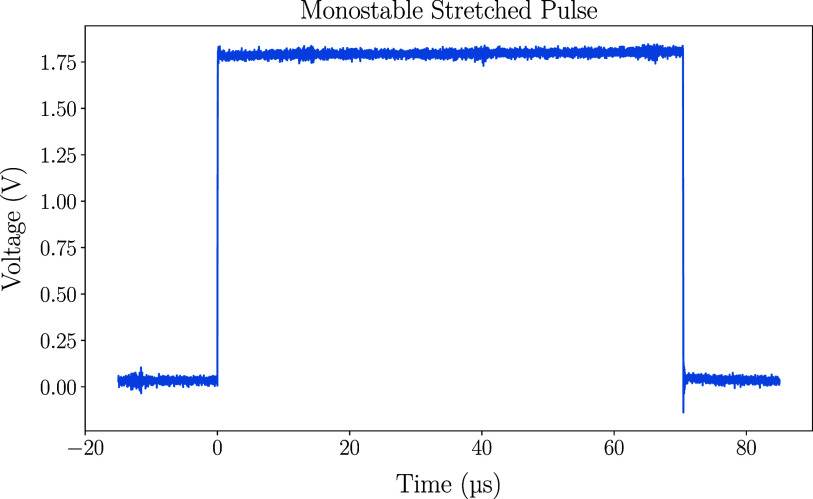
Monostable pulse stretcher output captured with the PicoScope. The SN74AHC123AN monostable produces an active-HIGH pulse of $\approx\!{68}\unicode{x03BC}\textrm{m}$ in response to each $\approx\!{200}\,\textrm{ns}$ trigger pulse from the ultrasound scanner. The pulse width is set by the external timing components $R = {10}{\,\textrm{k}\Omega}$ and $C = {6.8}\,\textrm{nF}$.

A monostable multivibrator (SN74AHC123AN, Texas Instruments Inc. Dallas, TX, USA) stretches the $\approx\!{200}\,\textrm{ns}$ input pulse into a $\approx\!{68}\unicode{x03BC}\textrm{m}$ output pulse; the circuit schematic is shown in figure 2(a) and the output waveform in figure 5. The output pulse width is set by an external timing resistor ($R = {10}{\,\textrm{k}\Omega}$) and capacitor ($C = {6.8}\,\textrm{nF}$) according to $T = KRC$, where $K = 1.0$ for $C \unicode{x2A7E} {1000}{\,\textrm{pF}}$ [21]. Its trigger-to-output propagation delay is small and fixed — of order tens of nanoseconds (the datasheet specifies $t_{\mathrm{PLH}} \unicode{x2A7D} {24}\,\textrm{ns}$ at ${3.3}{\,\textrm{V}}$ and $\unicode{x2A7D} {14}\,\textrm{ns}$ at ${5}{\,\textrm{V}}$ VCC for a ${15}{\,\textrm{pF}}$ load [21]).

The 74AHC123’s input thresholds scale linearly with its supply voltage [21]: $V_{\mathrm{IH}} \approx 0.7\,\mathrm{VCC}$ and $V_{\mathrm{IL}} \approx 0.3\,\mathrm{VCC}$. At ${5}{\,\textrm{V}}$ VCC, the thresholds ($V_{\mathrm{IH}} = {3.5}{\,\textrm{V}}$, $V_{\mathrm{IL}} = {1.5}{\,\textrm{V}}$) would leave the ultrasound’s ${1.6}{\,\textrm{V}}$ idle level in the undefined zone between them, preventing reliable detection. The monostable is therefore powered at ${2}{\,\textrm{V}}$ VCC, generated by a resistive divider from the Arduino’s ${5}{\,\textrm{V}}$ rail. At this supply, the thresholds drop to $V_{\mathrm{IH}} = {1.4}{\,\textrm{V}}$ and $V_{\mathrm{IL}} = {0.6}{\,\textrm{V}}$, placing the ${1.6}{\,\textrm{V}}$ idle level comfortably above $V_{\mathrm{IH}}$ and the ${0}{\,\textrm{V}}$ active level well below $V_{\mathrm{IL}}$.

A ${50}\,{\Omega}$ terminator at the scanner output and a ${220}\,\,{\Omega}$ series resistor at the chain entry suppress BNC cable ringing, and a ${100}\,\textrm{nF}$ ceramic capacitor decouples VCC.

#### 2.2.2. Logic level shifter

**Figure 6.**
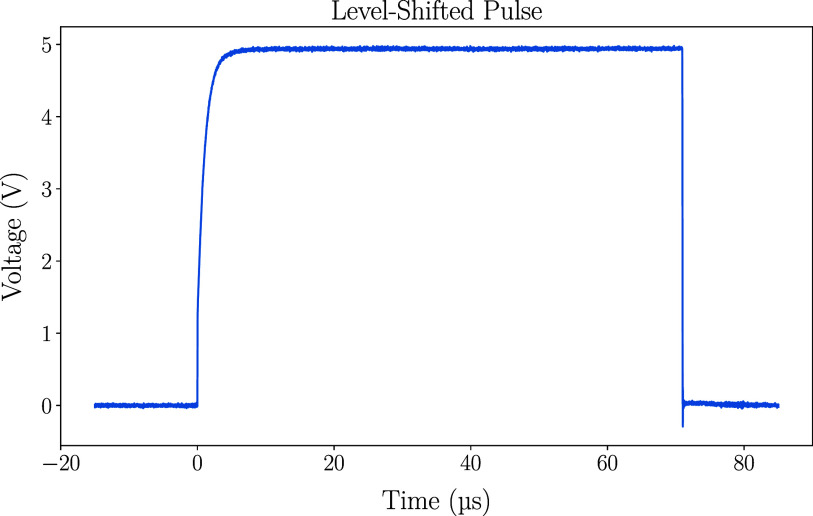
Logic level shifter output captured with the PicoScope. The Pololu #2595 bidirectional level shifter converts the monostable’s 0–2 V pulse to the 0–5 V levels required by the Arduino’s D2 (INT0) input.

A bidirectional MOSFET-based level shifter (Pololu #2595) [22] converts the monostable’s 0–2 V output to 0–5 V for the Arduino; the circuit schematic is shown in figure 2(b) and the output waveform in figure 6. The low-voltage reference is connected to the same ${2}{\,\textrm{V}}$ tap that powers the monostable; the high-voltage reference is the Arduino’s ${5}{\,\textrm{V}}$ rail. Its rising edge is RC-limited by the ${10}{\,\textrm{k}\Omega}$ pull-up; the passive MOSFET design is rated for ${400}\,\textrm{kHz}$ fast-mode $I^2C$, i.e. a rise time $ < {300}\,\textrm{ns}$ [22]. The level-shifted output connects to pin D2 on the Arduino (hardware interrupt INT0), configured for rising-edge detection.

### 2.3. Firmware

**Figure 7.**
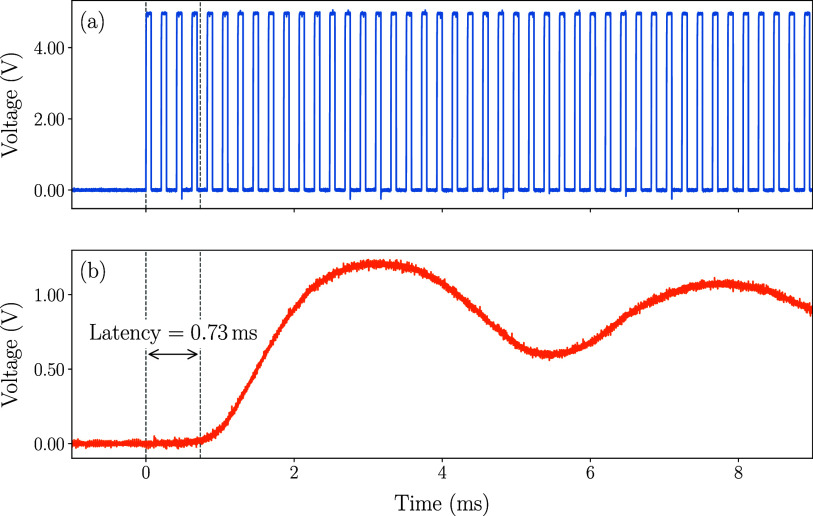
Representative single-trial PicoScope capture of the device latency. (a) The train of conditioned trigger pulses on pin D2; the rising edge of the first pulse defines $t = 0$. (b) The output of a FlexiForce A301 force sensor placed under the MPPV-2 piston. The vertical dashed lines mark the detected onset of the force response relative to $t = 0$. The oscillation of the FlexiForce signal reflects the mechanical step response.

The LOGIQ^®^ E10 trigger output emits a pulse at every beam firing, producing a continuous train of triggers during a cine loop (figure 7(a)). To insert a single fiducial mark per cine loop, the firmware uses an arm/disarm mechanism: the operator arms the system via serial command, the firmware fires once on the next trigger, and disarms automatically. On each armed trigger, the firmware executes the following sequence:

1. **Trigger detection**: the firmware detects the trigger only if the system is armed.
2. **Advance**: the firmware sets the direction pin HIGH, enables the motor, and issues $N$ step pulses at 500 steps/s (2 ms/step).
3. **Dwell**: the piston remains at peak displacement for ${200}\,\textrm{ms}$ to ensure the receiver registers the sustained event.
4. **Retract**: the firmware sets the direction pin LOW and issues $N$ step pulses to return the piston to its rest position.
5. **Disable**: the ENABLE pin is driven LOW to de-energize the motor.
6. **Disarm**: the system returns to the disarmed state.

Before each acquisition, the operator pre-positions the piston into contact with the catheter and arms the system; the firmware then waits for the next trigger pulse, fires once, and disarms.

Besides this single-shot mode, the firmware can fire repeatedly, emitting one marker every preset number of triggers to place multiple markers in a single recording (as in the characterization run below, section 2.5). More generally, because the device fires a marker on any source trigger, two or more markers spanning a recording measure the source–receiver clock drift directly, for any source firing below the $\approx\!{15}\,\textrm{kHz}$ rate at which the stretcher’s $\approx\!{68}\unicode{x03BC}\textrm{m}$ output pulses begin to merge.

### 2.4. Device-side timing characterization

We define the device latency as the interval across the device — from the conditioned trigger edge at the Arduino input (pin D2) to the onset of the actuator’s mechanical response. We measure it with the PicoScope as the delay from the D2 pulse to the output of a FlexiForce A301 piezoresistive force sensor (Tekscan, South Boston, MA, USA; response time $ < {5}\,\,\unicode{x03BC}\textrm{m}$ [23]) placed between the MPPV-2 piston and a rigid backing surface (figure 2(d)). The device latency comprises two stages: the Arduino firmware and the electromechanical response of the stepper driver and actuator. To separate them, we also measure an Arduino-only latency — the same D2 signal against the first step pulse leaving pin D5 — and take the electromechanical component as the difference. We collected $n = 30$ trials of each measurement.

The upstream signal conditioning (BNC, pulse stretcher, level shifter) precedes the D2 measurement window and is therefore not included in the device latency. It adds only a fixed delay of well under ${1}\unicode{x03BC}\textrm{m}$ — the BNC cable’s few-nanosecond transit, the stretcher’s tens-of-nanosecond propagation delay, plus the level shifter’s ${ < }{300}\,\textrm{ns}$ rise time — and contributes negligible trial-to-trial jitter. This fixed delay is far smaller than the device latency, and its jitter far smaller than the device jitter, so excluding this stage does not affect the characterization.

Per-trial latencies were computed as the time from the trigger rising edge at D2 to the onset of the response signal, using our in-house onset detector [24, 25]. Briefly, the detector locates the response peak and estimates a robust baseline — a median level and a median absolute deviation scatter — from the flat, pre-rise portion of the window. It sets a signal threshold at a fixed number of standard deviations above the baseline median and a companion threshold on the signal’s time-derivative, then scans back from the peak and marks the onset as the earliest sample that both stay above the signal threshold and is still actively rising (its time-derivative above the companion threshold). This derivative condition keeps an elevated but flat part of the trace from being mistaken for the rising edge. The onset is reported at the native sample resolution, without sub-sample interpolation.

### 2.5. Ultrasound–HRM timing characterization

We recorded a firing sequence with an HRM (ManoScan ESO, Medtronic, Minneapolis, MN, USA) catheter (2.75 mm in diameter) loaded in the actuator, with the firing driven by the ultrasound scanner: the firmware fired a marker every ${48000}$ beam triggers (an arbitrary large count, set only to space successive markers $\approx\!{17}\,\textrm{s}$ apart. — far beyond the actuator’s advance–dwell–retract cycle) and recorded each marker’s beam index. This produced $n = 30$ markers over an $\approx\!{8}\,\textrm{min}$ acquisition. The actuator preloaded the catheter by advancing $20$ steps ($\approx\!{0.25}\,\textrm{mm}$); each firing then advanced $10$ steps ($\approx\!{0.13}\,\textrm{mm}$) as the marker, dwelled for ${200}\,\textrm{ms}$, and retracted, and the markers were recorded by the HRM at its native ${100}\,\textrm{Hz}$ sample rate. It is worth noting that the ManoScan ESO carries $36$ pressure sensors along the catheter, and the marker is registered by the single sensor beneath the piston. In the intended clinical use this is one of the external sensors — only about $10$ of the $36$ lie inside the body — so the marker does not share a sensor with the in-body physiological measurement [26].

The ultrasound time of each marker was taken from the scanner’s own clock rather than an external stopwatch, so the comparison remains ultrasound-versus-HRM: the cine’s DICOM metadata records the per-frame time and the number of transmit beams per frame, whose ratio is the inter-beam interval $\tau$ (here $\tau = {349}\unicode{x03BC}\textrm{m}$); a marker fired at beam $b$ then has ultrasound time $t_{\mathrm{US}} = b\,\tau$. We located each marker’s rising-edge onset with the same in-house onset detector used for the device-side measurements (section 2.4) and fitted the detected onsets against ultrasound time with a free two-parameter line. The slope of this line is unity in an ideal drift-free case, and its departure from one is the ultrasound–HRM clock drift; the residual scatter about the line is the per-marker jitter that limits alignment precision (both quantified in section 3.2). In production, the first trigger fires a marker on the first beam of the cine loop, fixing cine-time-zero on the HRM axis (after subtracting the fixed, calibrated device latency); the known frame rate then places every ultrasound frame relative to it. If clock drift over the loop is a concern, a second marker at the last beam can be added to bracket the acquisition, allowing the drift to be measured and removed.

## 3. Results and discussion

### 3.1. Device-side timing

**Figure 8.**
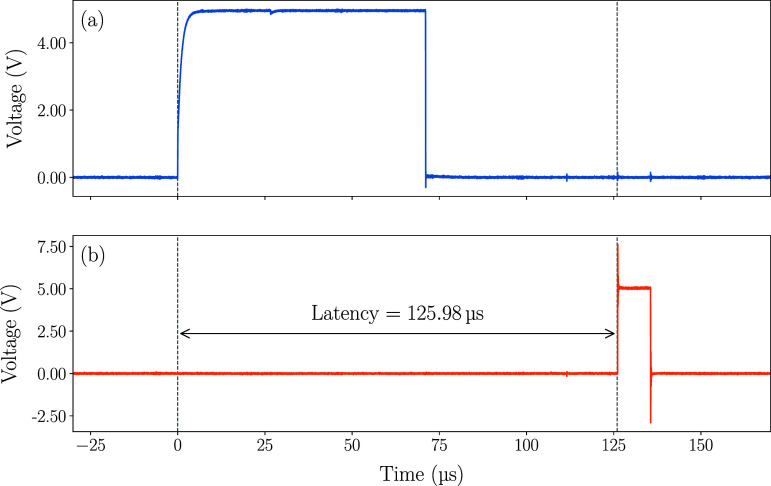
Representative single-trial PicoScope capture isolating the Arduino-only latency. (a) Conditioned trigger pulse arriving at pin D2; the rising edge defines $t = 0$. (b) First step pulse leaving pin D5 to the DRV8434A driver. The vertical dashed line marks the detected onset of the D5 pulse relative to $t = 0$. The probe-tip ringing in panel (b) is a measurement artifact from the clip-lead ground and does not affect the onset determination.

**Figure 9.**
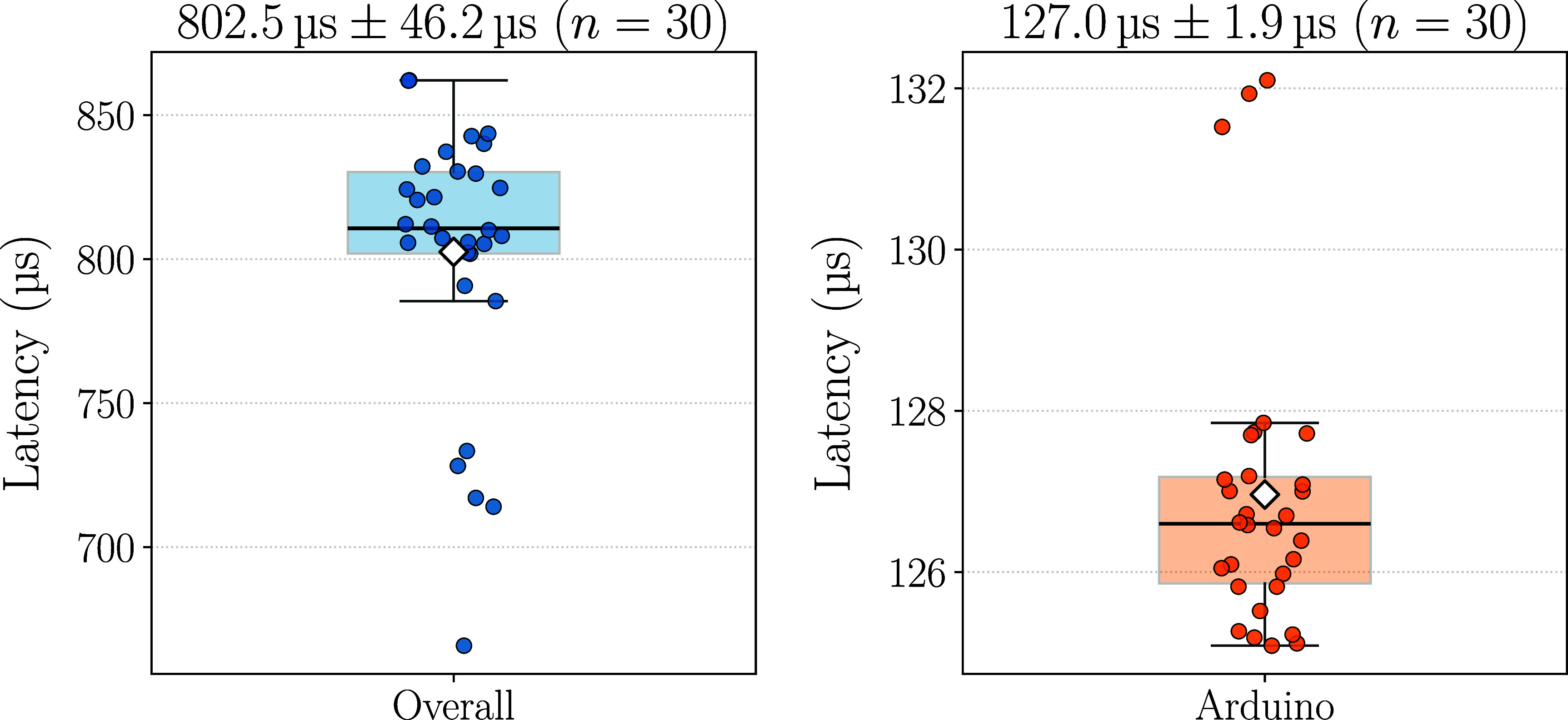
Per-trial distributions of the device latency (left, blue) and the Arduino-only latency (right, orange) across $n = 30$ trials each. Each panel shows every trial as a point, a box plot summarizing the median and interquartile range (with whiskers at $1.5 \times$ IQR), and a white diamond marking the mean. Panel titles report mean $\pm$ SD in microseconds.

The device-side timing characteristics are shown in figures 7–9. More specifically, single-trial captures of the device-latency and Arduino-only measurements appear in figures 7 and 8, respectively. The per-trial distributions across $n = 30$ trials of each series (figure 9, table 1) gave a device latency of ${802.5}\unicode{x03BC}\textrm{m} \pm {46.2}\unicode{x03BC}\textrm{m}$ (mean $\pm$ SD; peak-to-peak range ${196.3}\unicode{x03BC}\textrm{m}$) and an Arduino-only latency of ${127.0}\unicode{x03BC}\textrm{m} \pm {1.9}\unicode{x03BC}\textrm{m}$ (peak-to-peak range ${7.0}\unicode{x03BC}\textrm{m}$). Subtracting the means and variances under the assumption of independence gives an estimated electromechanical latency of $\approx\!{675.5}\unicode{x03BC}\textrm{m} \pm {46.2}\unicode{x03BC}\textrm{m}$, accounting for $\approx\!{84}\%$ of the device latency and the dominant share of the trial-to-trial variation: the Arduino-only variance is only $\approx\!{0.2}\%$ of the device-latency variance.

**Table 1.** Per-trial latency statistics for the device-side measurements ($n = 30$ trials each) and per-marker residual statistics for the HRM-side measurement ($n = 30$ markers). Bracketed values in the Mean and SD columns are 95% confidence intervals.

Series	$n$	Mean	SD	Min	Max	Range
Device-side timing ($\unicode{x03BC}\textrm{s}$)						

Device latency	30	802.5 [785.3, 819.8]	46.2 [36.8, 62.1]	665.8	862.1	196.3
Arduino-only	30	127.0 [126.3, 127.7]	1.9 [1.5, 2.5]	125.1	132.1	7.0
Electromechanical$ ^{\textrm{a}}$	—	675.5 [658.3, 692.8]	46.2 [36.8, 62.1]	—	—	—

HRM-side timing (ms)						

Per-marker residuals$ ^{\textrm{b}}$	30	—	6.36 [5.07, 8.55]	—	—	25.2

$ ^\textrm{a}$ Derived from the device-latency and Arduino-only series under the assumption of independent errors: $\mu_{\mathrm{mech}} = \mu_{\mathrm{device}} - \mu_{\mathrm{Arduino}}$ and $\sigma_{\mathrm{mech}}^2 = \sigma_{\mathrm{device}}^2 - \sigma_{\mathrm{Arduino}}^2$. Min, max, and range are not defined for this derived quantity because the two series were captured in independent PicoScope runs and cannot be paired trial-by-trial.

$ ^\textrm{b}$ Residuals are deviations from a free two-parameter linear fit to the marker onsets (section 2.5); the least-squares fit makes their mean zero by construction, so only the spread (SD and range) is reported as the per-marker jitter.

### 3.2. Ultrasound–HRM timing

**Figure 10.**
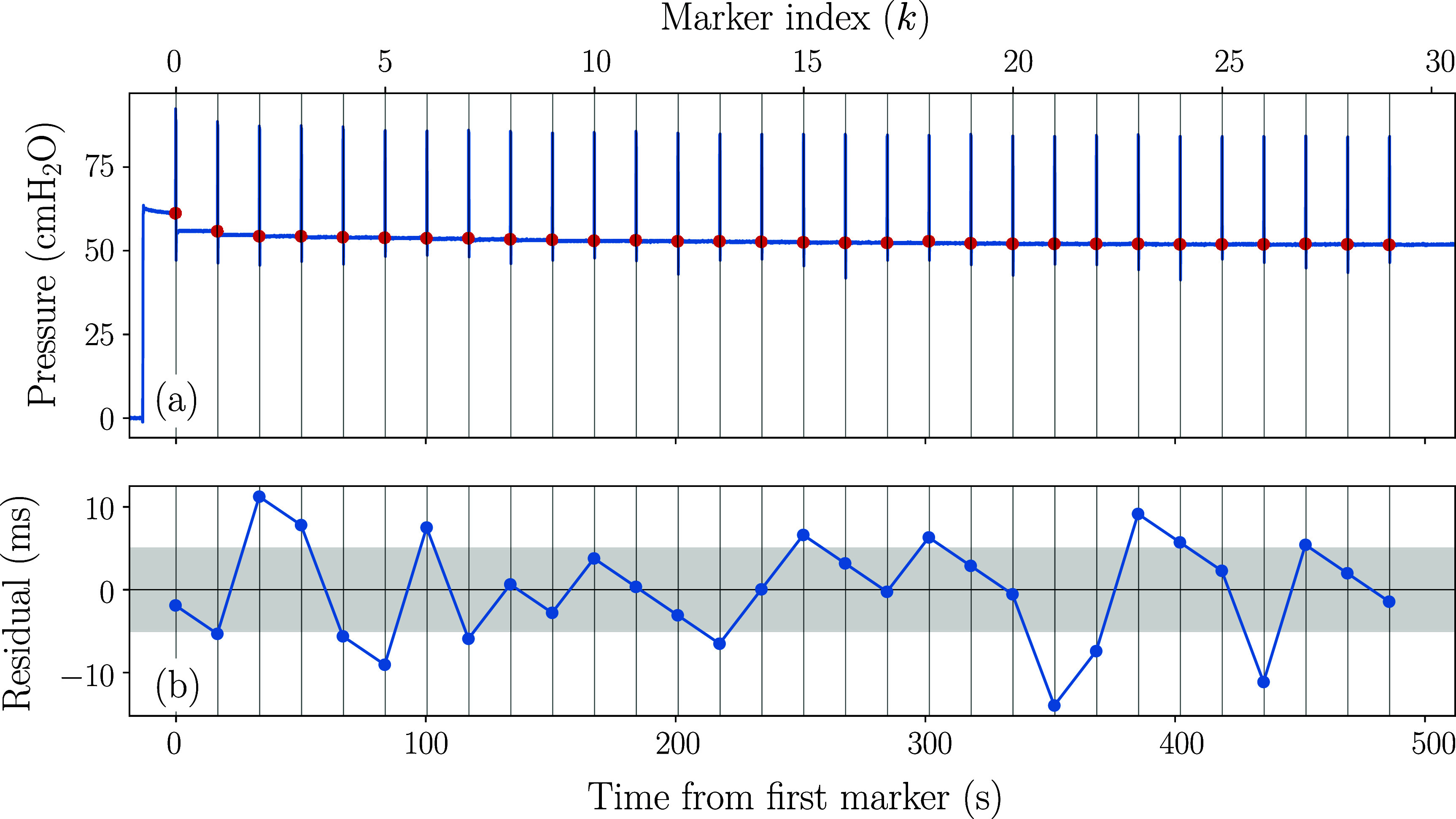
Ultrasound–HRM characterization run. The ultrasound scanner drove $n = 30$ markers (one every ${48000}$ beam triggers) with an HRM catheter loaded in the actuator slot. (a) Full HRM pressure trace (blue) with the detected marker onsets overlaid (red dots) and the expected marker-time grid (faint black vertical lines, $\approx\!{17}\,\textrm{s}$ apart); the secondary $x$-axis labels the marker index. (b) Per-marker residuals about the free linear fit, plotted versus time, with the $\pm \mathrm{d}t/2 = \pm {5}\,\textrm{ms}$ sample-quantization band shaded in gray.

**Figure 11.**
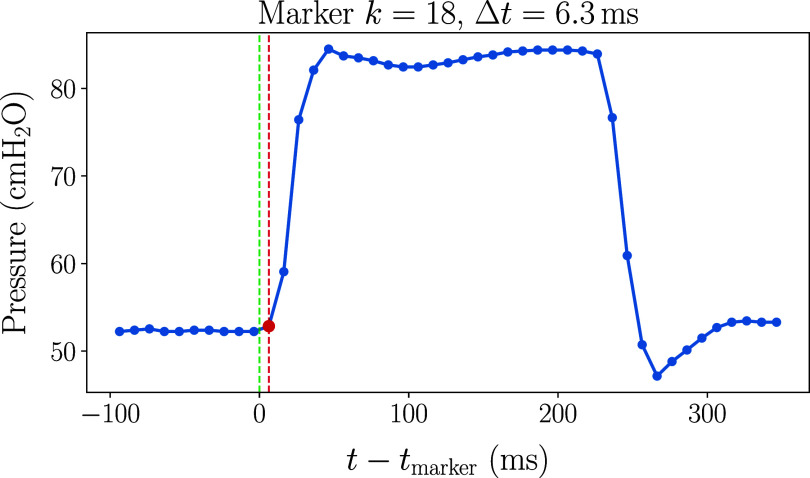
Representative single marker from the characterization run, zoomed to the millisecond scale (marker index $k = 18$). The pressure trace (blue line, with samples shown as dots to make the ${10}\,\textrm{ms}$ HRM sampling interval visible) is plotted against time relative to the marker. The green dashed vertical marks the expected marker time and the red dashed vertical marks the detected onset.

**Figure 12.**
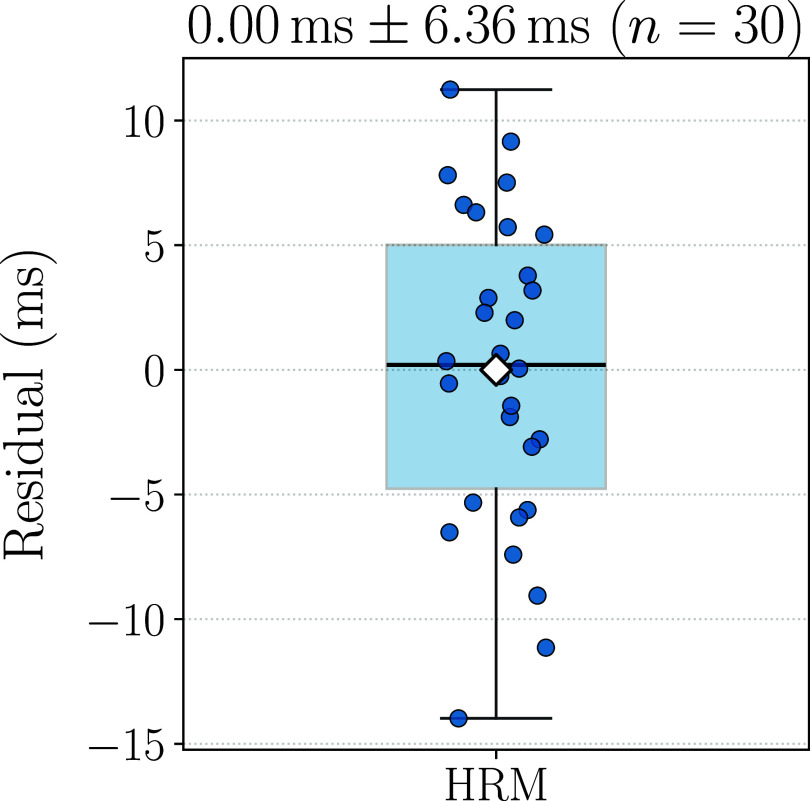
Per-marker residual distribution from the ultrasound–HRM characterization run ($n = 30$ markers). Each residual is shown as a point, the box summarizes the median and interquartile range (whiskers at $1.5 \times$ IQR), and the white diamond marks the mean. The panel title reports mean $\pm$ SD.

The HRM-side timing characteristics are shown in figures 10–12: the full HRM pressure trace with the detected marker onsets and per-marker residuals (figure 10), and a single marker zoomed to the millisecond scale (figure 11). Over the $\approx\!{8}\,\textrm{min}$ run, all $30$ markers fired and were detected, with no missed or spurious firings. The per-marker residual distribution across $n = 30$ markers (figure 12, table 1) gave a standard deviation of $\sigma_{\mathrm{HRM}} = {6.36}\,\textrm{ms}$ (peak-to-peak range ${25.2}\,\textrm{ms}$). This $\sigma_{\mathrm{HRM}}$ reflects the HRM’s ${100}\,\textrm{Hz}$ sample rate (sample interval $\Delta t = {10}\,\textrm{ms}$; max quantization error $\Delta t/2 = {5}\,\textrm{ms}$). A timestamp returned at sample resolution carries a uniform quantization error of variance $\Delta t^2/12$, ^6^6The variance of a uniform distribution on an interval of length $w$ is $w^2/12$, obtained by integrating $x^2$ against the uniform density. with SD $\Delta t/\sqrt{12} \approx {2.89}\,\textrm{ms}$ — the theoretical floor for $\sigma_{\mathrm{HRM}}$. The empirical ${6.36}\,\textrm{ms}$ exceeds this floor in part because the onset detector returns sample-resolution timestamps rather than sub-sample-interpolated ones, and in part because the HRM transducer’s response time of $\approx\!{25}\,\textrm{ms}$ blurs the pressure rise across multiple samples, broadening the onset distribution beyond what sub-sample interpolation alone could recover.

**Figure 13.**
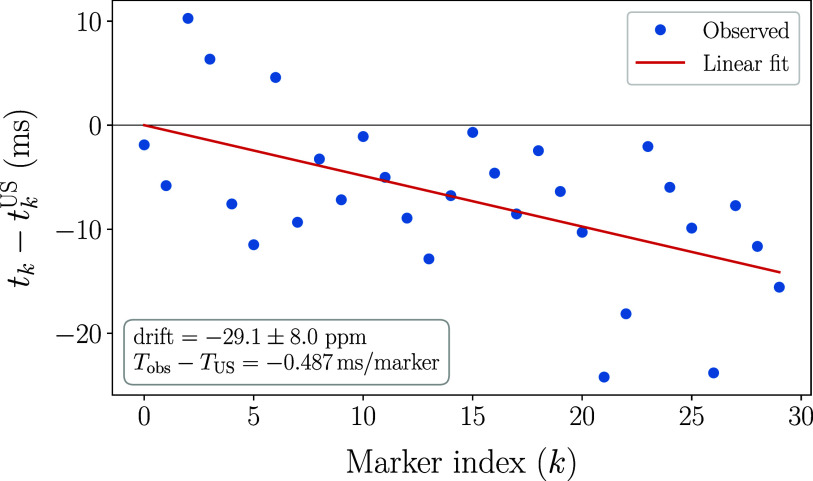
Ultrasound–HRM clock-drift measurement ($n = 30$ markers). Each point is the deviation of a detected marker onset from its ultrasound-reported time, plotted against marker index; the slope of the linear fit, normalized by the inter-marker interval, is the ultrasound–HRM clock drift, $-29 \pm {8}\,\textrm{ppm}$.

The ultrasound–HRM clock drift — the departure of the fitted slope from unity (section 2.5) — is $-29 \pm {8}\,\textrm{ppm}$ (figure 13), with the markers driven by the ultrasound trigger and the timebase read from the cine’s DICOM frame timing. Over a ${10}\,\textrm{s}$ cine loop, $-$29 ppm accumulates to only $\approx\!{0.3}\,\textrm{ms}$ — far below both $\sigma_{\mathrm{HRM}}$ (${6.36}\,\textrm{ms}$) and a single frame interval — so drift is negligible for the demonstrated application.

**Figure 14.**
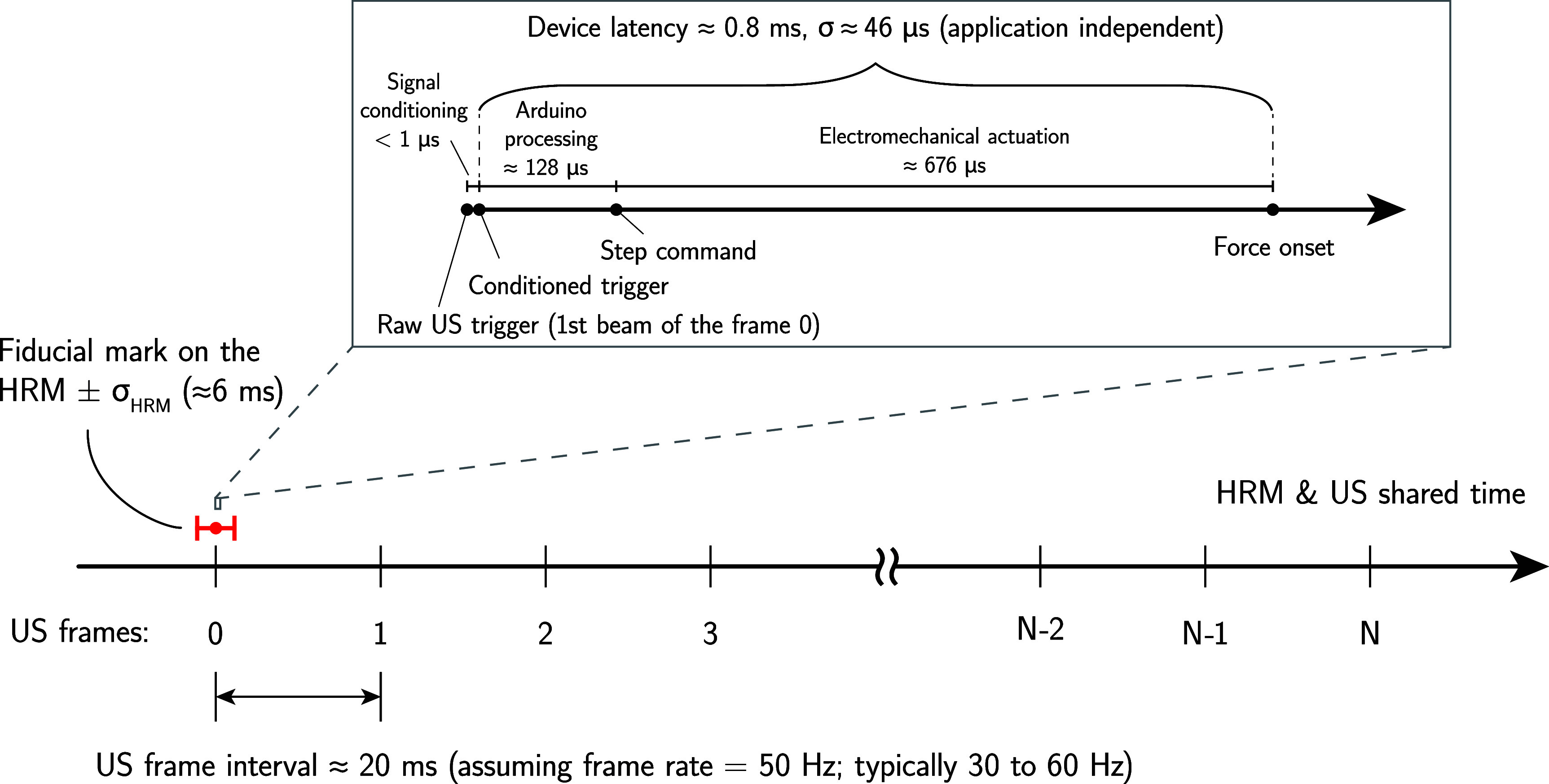
Timing diagram of the synchronization scheme: the raw ultrasound trigger (the first beam of cine frame 0) is conditioned, processed by the Arduino, and actuated into a fiducial mark on the HRM trace, anchoring the HRM recording to the ultrasound frame-index axis (bottom). The device latency (inset; $\approx\!{0.8}\,\textrm{ms}$, application-independent) is a fixed offset far smaller than the ultrasound frame interval, and its jitter ($\sigma \approx {46}\unicode{x03BC}\textrm{m}$) is far below $\sigma_{\mathrm{HRM}}$, so the device limits neither the frame assignment nor the synchronization uncertainty. Time intervals are schematic, drawn to qualitative rather than quantitative scale.

The full timing chain and budget are summarized in figure 14. Only two terms carry trial-to-trial (random) uncertainty — the device jitter $\sigma_{\mathrm{device}} = {46.2}\unicode{x03BC}\textrm{m}$ (section 3.1) and the HRM-side jitter $\sigma_{\mathrm{HRM}} = {6.36}\,\textrm{ms}$. Under the assumption of independent errors, they combine to $\sigma_{\mathrm{total}} = \sqrt{\sigma_{\mathrm{device}}^2 + \sigma_{\mathrm{HRM}}^2} \approx {6.36}\,\textrm{ms}$, indistinguishable from $\sigma_{\mathrm{HRM}}$ itself ($\sigma_{\mathrm{device}} / \sigma_{\mathrm{HRM}} \approx {0.7}\%$). The device therefore does not limit synchronization accuracy for this application. This conclusion is robust to the finite sample size: even combining the upper $95\%$ bound on $\sigma_{\mathrm{device}}$ (${62.1}\unicode{x03BC}\textrm{m}$, table 1) with the lower bound on $\sigma_{\mathrm{HRM}}$ (${5.07}\,\textrm{ms}$), the ratio $\sigma_{\mathrm{device}} / \sigma_{\mathrm{HRM}}$ stays $\approx\!{1}\%$. Every other term in the end-to-end timing chain (figure 14) adds nothing further to this uncertainty — each is either a fixed offset that is calibrated out, exact by construction, negligible, or already absorbed into $\sigma_{\mathrm{HRM}}$:

• the device latency ($\approx\!{0.8}\,\textrm{ms}$, dominated by the electromechanical stage) is a fixed, calibrated systematic offset that is subtracted when the marker is placed on the HRM axis (section 2.5)
• the signal-conditioning delay is fixed, with jitter $\ll{1}\unicode{x03BC}\textrm{m}$ (sections 2.2 and 2.4)
• the trigger-to-frame mapping is exact by construction, since the first trigger fires the marker on the first beam of frame 0 (section 2.5)
• the clock drift is negligible ($\approx\!{-29}\,\,\textrm{ppm}$)
• the onset-detection and HRM sensor-response variability are not separate terms but are already part of $\sigma_{\mathrm{HRM}}$, which is measured from the onset distribution they broaden

This $\approx\!{6}\,\textrm{ms}$ uncertainty is small relative to both the ultrasound cine-loop frame interval (typically 17–33 ms at 30–60 Hz) and the bandwidth of the pressure waveforms of interest (which, for example, have most power below $\approx\!{20}\,\textrm{Hz}$ during a cough [27]); the alignment error is well below a single frame interval, so each HRM pressure event can be unambiguously matched to its corresponding ultrasound frame.

### 3.3. Limitations

This work introduces the approach and characterizes the synchronization device; several limitations remain. The synchronization uncertainty is set jointly by the source, the device, and the receiver — which of the three dominates depends on the pairing — so it must be characterized anew for each one: for the HRM here the receiver dominates, through its sample rate, sensor response time, and onset-detection method (section 3.2). The marker also depends on the mechanical coupling between the piston and the receiver, which varies with catheter type and how it seats in the actuator; its amplitude must be chosen large enough to be detected reliably yet small enough not to damage the catheter (here a $10$-step, $\approx\!{0.13}\,\textrm{mm}$ advance). We characterized the device on the bench in a single, fixed configuration; reproducibility across sessions, power cycles, preload settings, actuator displacements, and catheter placements — and the associated run-to-run variability — was not assessed and is left to a broader validation study. The signal-conditioning front end is likewise a bench prototype: the ${2}{\,\textrm{V}}$ logic rail is derived from the Arduino’s ${5}{\,\textrm{V}}$ supply through a resistive divider. This sufficed for the bench characterization reported here, but a production unit would replace it with a regulated reference or a comparator/Schmitt-trigger front end. Finally, although the approach applies in principle to any mechanically-sensitive receiver of adequate bandwidth, we demonstrate it only for HRM; extension to other receivers (e.g. strain gauges, accelerometers, piezoelectric sensors) requires the analogous receiver-side timing characterization.

## 4. Conclusions

We described and characterized a mechanically-induced fiducial marker device that synchronizes independent recording instruments by converting an electrical trigger from one instrument into a brief mechanical event sensed by the other. The device’s intrinsic jitter of ${46.2}\unicode{x03BC}\textrm{m}$ is the lower bound on synchronization uncertainty for any application; the actual achievable uncertainty depends on the receiver’s timing precision (its sample rate, onset-detection method, and sensor response characteristics), which must be characterized analogously to the HRM-side measurement reported here. For the ultrasound–HRM pairing demonstrated, the HRM-side jitter dominates ($\sigma_{\mathrm{HRM}} \approx {6}\,\textrm{ms}$), so the synchronization uncertainty is set by the HRM sample interval; for receivers with sample rates substantially higher than the HRM’s ${100}\,\textrm{Hz}$, the device’s intrinsic jitter approaches the dominant contribution. The design pattern applies to instrument pairings that satisfy four conditions: a stable electrical trigger, a known mapping from that trigger to the source’s timeline, a mechanically-sensitive receiver of adequate bandwidth, and a marker that does not contaminate the signal of interest.

## Data Availability

The data cannot be made publicly available upon publication because the cost of preparing, depositing and hosting the data would be prohibitive within the terms of this research project. The data that support the findings of this study are available upon reasonable request from the authors.
